# Factors influencing pregnant women’s use of antenatal and emergency care services covered by social security: findings from the maternal eCohort in Mexico

**DOI:** 10.1186/s12884-025-08301-9

**Published:** 2025-11-04

**Authors:** Svetlana V. Doubova, Martín Paredes Cruz, Diana Perez-Moran, Luis Rey Garcia Cortes, Gilberto Espinoza Anrubio, Claudia Elsa Perez Ruiz, Megan Carolina Cerda Mancillas, Carlos Alberto Prado Aguilar, Miguel Ángel Romero Garcia, Augusto Sarralde Delgado, Ricardo Pérez-Cuevas, Catherine Arsenault, Claudio Quinzaños Fresnedo

**Affiliations:** 1 Unidad de Investigación Epidemiológica y Servicios de Salud del CMN SXXI, Instituto Mexicano del Seguro Social, Av. Cuauhtemoc 330, Ciudad de México, 06720 México; 2 OOAD Estado de México Oriente, Instituto Mexicano del Seguro Social, Estado de México, Av. Recursos Hidráulicos 2 A, Fracc. Industrial La Loma, Tlalnepantla de Baz, Mexico 54060 México; 3 OOAD DF Sur. Instituto Mexicano del Seguro Social, Alcaldía Iztapalapa Ciudad de México, Calzada de la Viga No 1174, Torre B, Piso 2. Colonia El Triunfo, Alcaldía Iztapalapa Ciudad de México, 09430 México; 4 OOAD Veracruz Norte, Instituto Mexicano del Seguro Social, Lomas del Estadio s/n, Col. Centro Xalapa de Enríquez, Veracruz, 91000 Mexico; 5 OOAD Coahuila, Instituto Mexicano del Seguro Social. Blvd. Venustiano Carranza, 2809. Colonia La Salle. , Saltillo, Coahuila 25280 México; 6 OOAD Aguascalientes, Instituto Mexicano del Seguro Social, Av. Alameda 707, Colonia del trabajo, Aguascalientes, 20180 México; 7 Jefatura de Prestaciones Médicas, Instituto Mexicano del Seguro Social, Condominio Monterrey 5º. Piso. Morelos 133, Centro, Monterrey, Nuevo León 64000 Mexico; 8 Jefatura de Servicios de Prestaciones Médicas. Coordinación Auxiliar Médica de Investigación en Salud. OOAD Jalisco, Instituto Mexicano del Seguro Social, Belisario Domínguez No. 1000. Col. Independencia , Guadalajara, Jalisco 44340 Mexico; 9 Division of Social Protection and Health, Interamerican Development Bank, 1300 New York Avenue, N.W. Washington, DC, 20577 USA; 10 Department of Global Health. Milken Institute School of Public Health, The George Washington University, Washington, DC, USA; 11 Dirección de Prestaciones Médicas, Instituto Mexicano del Seguro Social, Colonia Roma Norte. Alcaldía Cuauhtémoc, Ciudad de México, 06700 México

**Keywords:** Antenatal care use, Emergency service utilization, Associated factors, Mexico

## Abstract

**Background:**

Evidence on antenatal care (ANC) and emergency service utilization during pregnancy in Latin America remains limited. Understanding these patterns is critical given the substantial benefits of ANC and emergency services in improving maternal and neonatal outcomes, preventing and managing pregnancy complications, and reducing maternal and infant mortality. This study examined ANC and emergency service utilization among pregnant women affiliated with the Mexican Institute of Social Security (IMSS) and identified factors associated with their use.

**Methods:**

We conducted an observational eCohort study through telephone interviews with 1,390 pregnant women aged 18–49 who initiated ANC at IMSS. Outcomes included the number of ANC visits, emergency room use, perceived quality of ANC, and provider competence assessed through 12 recommended ANC activities: blood pressure and weight measurement, blood and urine tests, ultrasound, and counseling on nutrition, warning signs, birth preparedness, newborn care, psychological health, and folic acid and iron supplementation. Multivariable negative binomial regression models were used to identify factors associated with ANC attendance and emergency service use.

**Results:**

Of the full cohort, 3.7% exited due to miscarriage, 12.4% dropped out after baseline, and 11.8% dropped out during follow-up. One-third of women who left rated the quality of their first ANC visit as poor or fair. In addition, 30% of women who remained in the cohort reported poor or fair ANC quality. On average, participants reported six ANC visits, with 64.8% receiving at least 80% of the 12 recommended ANC activities. Nearly 30% sought emergency services during pregnancy. Higher ANC attendance was associated with hypertension, urinary infections, anemia, early ANC initiation, consultations with obstetricians, and hospital-based ANC follow-ups. Use of emergency services was more common among women with chronic diseases, hypertensive disorders of pregnancy, urinary tract infections, risk of miscarriage, or risk of depression.

**Conclusions:**

Substantial improvements in ANC are needed to strengthen women’s care experiences and promote consistent utilization of ANC services.

**Supplementary Information:**

The online version contains supplementary material available at 10.1186/s12884-025-08301-9.

## Background

Continuous, high-quality antenatal care (ANC) is an important channel for pregnant women’s health education and monitoring and is strongly linked to better health outcomes for both mothers and newborns [1–4]. Despite these benefits, ANC attendance in low—and middle-income countries is often inconsistent [5], heightening the risk of preventable complications [1].

ANC utilization is a complex behavior influenced by individual and contextual characteristics that can predispose, enable, or hinder healthcare services utilization [6]. Individual factors, such as being married, employed, having higher education, belonging to large families, residing in urban areas, being exposed to mass media, and having a history of miscarriage are associated with a greater likelihood of seeking ANC and attending more visits [5, 7–9]. In contrast, higher parity, unplanned pregnancy, and spousal opposition are barriers to ANC utilization. By comparison, contextual factors such as access to health insurance or public health services, proximity to a health facility, extended service hours, shorter waiting times, and perceived quality care of care, are associated with greater ANC use [10]. However, much of this evidence is drawn from studies conducted in Asia and Africa [5–9].

Additionally, some studies report high rates of emergency department (ED) use among pregnant women [11, 12]. While some of these visits are necessary, many are non-urgent and could be avoided with regular ANC [12]. Utilization of ED for primary care sensitive conditions, such as ANC-related issues, indicates gaps in primary care performance [13, 14]. Seeking treatment in ED for conditions that can be managed by primary care is costly [15], increases the risk of healthcare-associated infections [16] and is less likely to ensure continuity and integration of care, often resulting in lower patient satisfaction [17].

Several factors contribute to the utilization of ED services by pregnant women. These include obstetric or medical complications such as hemorrhage and hypertension and sociodemographic conditions like knowledge of pregnancy danger signs, lack of health insurance, domestic violence, and poverty [18–21]. Additionally, challenges related to health systems, such as personnel shortages and limited access to primary care, also play an important role [18–21]. Despite existing evidence, comprehensive data on ED use during pregnancy in low- and middle-income countries remain scarce, with most available research originating from high-income settings and many LMIC studies being qualitative [11, 12, 18–22].

In Latin America (LA), ANC coverage has increased significantly over the past two decades, with at least one ANC visit reported by 90% of women in most countries [23–26]. Despite this progress, maternal and infant mortality rates in many LA countries remain higher than the averages for members of the Organisation for Economic Co-operation and Development (OECD). For instance, in 2021 Mexico’s maternal mortality ratio was 53 deaths per 100,000 live births, compared with an OECD average of 10.9; infant mortality in Mexico was 12.7 per 1,000 live births, compared with 4.0 in the OECD [27–29]. Although the WHO 2016 recommendations [30] call for at least eight ANC contacts with a focus on prevention, timely detection, and counseling on maternal and newborn care, substantial variation exists both between and within LA countries. In Mexico, the Ministry of Health and other public health institutions define adequate ANC as a minimum of five visits. Significant gaps persist in ANC timing and content [31, 32]. According to the 2022 Mexican Health and Nutrition Survey (ENSANUT), only 62.6% of women initiated ANC by the eighth week of gestation, and the coverage of clinical activities varied widely—for example, 72% received counseling on physical activity during pregnancy, while 90.2% received folic acid supplementation [33]. Most ANC data in Mexico are collected via cross-sectional surveys asking about pregnancies in the past two years, which may introduce recall bias. A prospective cohort design could yield more precise, timely, and unbiased information on ANC and emergency care use.

To address this need, we conducted an observational maternal eCohort study at the Instituto Mexicano del Seguro Social (IMSS) across eight Mexican states, assessing women’s reported experiences of care from the first ANC visit through delivery and postnatal care [34].

IMSS is Mexico’s largest social health insurance provider, with 74 million affiliated members (nearly 60% of the country’s population), mainly formal labor market workers and their families. The institution offers primary, secondary, and tertiary health care through a nationwide network of 1,537 family medicine clinics (FMCs), 286 general hospitals, and 25 high-specialty hospitals [35]. IMSS provides a continuum of ANC and obstetric services in FMCs and hospitals. In FMCs, family physicians and nurses conduct ANC, assess obstetric risks, perform tests, administer vaccines, provide counseling on pregnancy-related topics (e.g., warning signs, nutrition, exercise, hygiene, breastfeeding), and monitor the pregnancy progress. In cases of complications, women are referred to obstetrician specialists at secondary and tertiary-level hospitals. At IMSS, ANC accounts for 3.1 million visits annually, making it the sixth most common reason for family medicine appointments. It is also the third leading cause of emergency service visits, with 1.2 million visits each year. The maternal eCohort included 1390 pregnant women enrolled between August and December 2023, with follow-up until August 2024 [34].

The present study analyzed patterns of ANC and emergency service utilization among pregnant women in the IMSS maternal eCohort and the factors associated with their use.

## Methods

This study analyzed data from the ANC follow-up of the observational maternal eCohort at IMSS. The eCohort consisted of 1,390 pregnant women aged 18–49 years, enrolled after their first ANC appointment with a family physician at primary family medicine clinics (FMCs). The study was conducted in 48 FMCs across eight Mexican states in four regions—North, West, Southeast, and Center—selected for their high ANC service volume. The states were Coahuila and Nuevo León (North), Aguascalientes and Jalisco (West), Veracruz and Yucatán (Southeast), and the State of Mexico and Mexico City (Central). From each state, two small, two medium, and two large FMCs were selected from the IMSS FMC registry, using the formula proposed by the Coordination of Information and Strategic Analysis and calculated as Total FMC affiliates/Total delegation affiliates x 100. Clinics with less than 5% of delegation affiliates were classified as small, 5 to 15% as medium, and more than 15% as large. Within each FMC, women were sampled using the Lahiri method [36] to enroll at least 1,300 women across regions. The sample size was estimated using the single population proportion formula, anticipating a 68% prevalence of low healthcare provider competence [37], 3% margin of error, 95% confidence, and 40% expected loss during follow-up. Detailed information on the eCohort inclusion criteria, sampling and sample size was previously published [34].

Data was collected with a validated ANC follow-up questionnaire designed by the Quality Evidence for Health System Transformation (QuEST) network, which assessed women’s perspectives on care quality during pregnancy through monthly telephone interviews. The questionnaire was adapted and validated by IMSS experts prior to data collection [34].

### Study variables

At baseline, data were collected on sociodemographic and clinical characteristics, and the content and quality of the first ANC visit. Monthly follow-up interviews recorded pregnancy-related health problems, ANC visit counts, content and quality and emergency room use.

The variables comprised:

1. General attributes that included (i) sociodemographic characteristics such as age, level of education, occupation, and marital status; (ii) risky health behaviors such as alcohol and tobacco consumption; and (iii) intimate partner violence (IPV) measured using the validated 19-item Mexican IPV scale [38], assessing psychological, physical, and sexual IPV. Presence of violence was defined as any affirmative response at baseline.
2. Obstetric and medical history covered parity (primigravida or multigravida), history of abortion or miscarriage, premature birth (less than 37 weeks of gestation); pre-gestational chronic conditions, such as thyroid disease, respiratory diseases, gastrointestinal diseases, gynecological illnesses, or other chronic conditions, including previously diagnosed diabetes, hypertension or cardiovascular disease.
3. Current pregnancy and health status included trimester of pregnancy at the first ANC visit, multiple pregnancies, and health problems presented during pregnancy, including hypertension or preeclampsia, gestational diabetes, anemia, threat of abortion, and urinary tract infections. The risk of depression was assessed at each interview using the Patient Health Questionnaire (PHQ-9), validated in Mexico [39, 40]. PHQ-9 scores range 0–27, categorized as minimal (0–4), mild (5–9), moderate (10–14), moderate–severe (15–19), severe (20–27) [41]. For this study, risk of depression was defined as a score ≥ 5 in one or more interviews during pregnancy.
4. Healthcare utilization during pregnancy comprised the number of ANC visits, emergency room visits, medical appointments for acute or chronic conditions, use of dental services, and the type of healthcare provider (IMSS only or visits to private providers as well), and consultations or follow-ups with specialists in obstetrics and gynecology. A visit to the emergency room was defined as an unscheduled presentation to a hospital or clinic’s emergency department by a woman seeking immediate medical evaluation and treatment, regardless of the urgency of the condition.
5. To assess the competence of ANC providers, we examined the completeness of twelve ANC activities recommended by IMSS experts. These activities included measuring blood pressure and weight at each ANC visit; referring to and informing patients of the results of blood and urine tests and ultrasound examinations at least once during ANC visits; counseling on nutrition, emergency signs recognition and response, preparing for birth and newborn care (e.g., hygiene, the first 6-month exclusive breastfeeding, importance and frequency of postnatal care), offering psychological counseling for those at risk of depression, administering folic acid supplementation during the first trimester and iron supplementation starting from 20 weeks of gestation. We then created a summative measure of care competence, defined as the percentage of twelve essential clinical actions the healthcare provider performed during ANC visits.
6. We also asked women to rate the quality of healthcare they received during each ANC visit on a scale from 1 to 5 (poor, fair, good, very good, and excellent). Due to a high variation in the number of ANC visits and varying perceptions of quality in each visit, we created a variable to indicate the perception of low quality when women reported poor or fair quality in one or more ANC visits.

The length of follow-up in the eCohort was determined by subtracting the gestational week when the woman began her ANC from the gestational week when she delivered. We defined 100% adherence to the follow-up as having one interview each follow-up month. 50% adherence was defined as having at least one interview every two months or more.

### Statistical analysis

We first described participant flow using frequencies and percentages. Second, to better understand if differences existed between the women who completed the survey and those who dropped out, we compared their baseline demographic characteristics, medical history, and first ANC visit attributes.

Third, to analyze health service use, we excluded 51 women with miscarriages from the analytical data set (46 with no ANC visits beyond the first, 5 with only 1–2 further visits). As a result, our final analytical sample included 1339 women. After that, we conducted a descriptive analysis of the study population and their use of health services. We summarized categorical variables using percentages, normally distributed numerical variables as mean (SD), and non-normally distributed variables as median (min–max).

Fourth, to address the primary objective of this manuscript, we analyzed factors associated with ANC and emergency service utilization. We performed two multivariable negative binomial regression models to account for overdispersion of both dependent variables. Our modeling strategy was based on VanderWeele and Shpitser’s criterion for confounder selection [42, 43], including all conceptually and clinically relevant covariates to ensure the final model adjusts for even slight confounding. We performed a literature review guided by the Andersen model of healthcare access and utilization [6] to identify relevant sociodemographic, obstetric, and medical history variables that previous studies linked to the use of ANC [5–10] and emergency services [18–21].

To address potential bias from the 12.4% dropout rate and additional missing data from 12 women, we applied stabilized inverse probability weights (IPW) when fitting the multivariable negative binomial regression models [44]. The denominator of stabilized IP weights was the probability of “having missing data” given the available covariates without missing data. These covariates were the participants’ age, occupation, presence of risky health behaviors, preexisting chronic disease, trimester of initiation of antenatal care, primigravida, and region of residence. The numerator was the probability of “having missing data” regardless of the covariates. The IP weights’ numerator and denominator were estimated using predicted probabilities from a logistic regression model.

Previous to multiple regression analysis, we verified absence of multicollinearity, (variance inflation factors < 5) and removed the variable “follow-up duration” due to collinearity with “ANC initiation trimester”. We also confirmed the absence of interactions among the following independent variables (1) “Suffering some type of intimate partner violence” and “Risk of depression during pregnancy”; (2) “ANC care perceived as good quality” and “ANC visits with private provider during pregnancy “; (3) “Single/divorced/separated” and “Suffering some type of intimate partner violence”, as none of the assessed potential interactions showed statistical significance.

In addition, standard errors of both regression models were adjusted for the clustered sampling design using the FMC as the cluster unit. Significance was set at *p* ≤ 0.05. Analyses were conducted using Stata 14 (StataCorp, College Station, TX). Reporting followed STROBE guidelines.

### Ethics

The study was approved by the IMSS National Research and Ethics Committees (R-2022-785−064). Before participating, all women were informed by trained interviewers about the study’s objectives, content, and voluntary nature, and they signed an informed consent form.

## Results

### Participant flow and follow-up rates

**Fig. 1:**
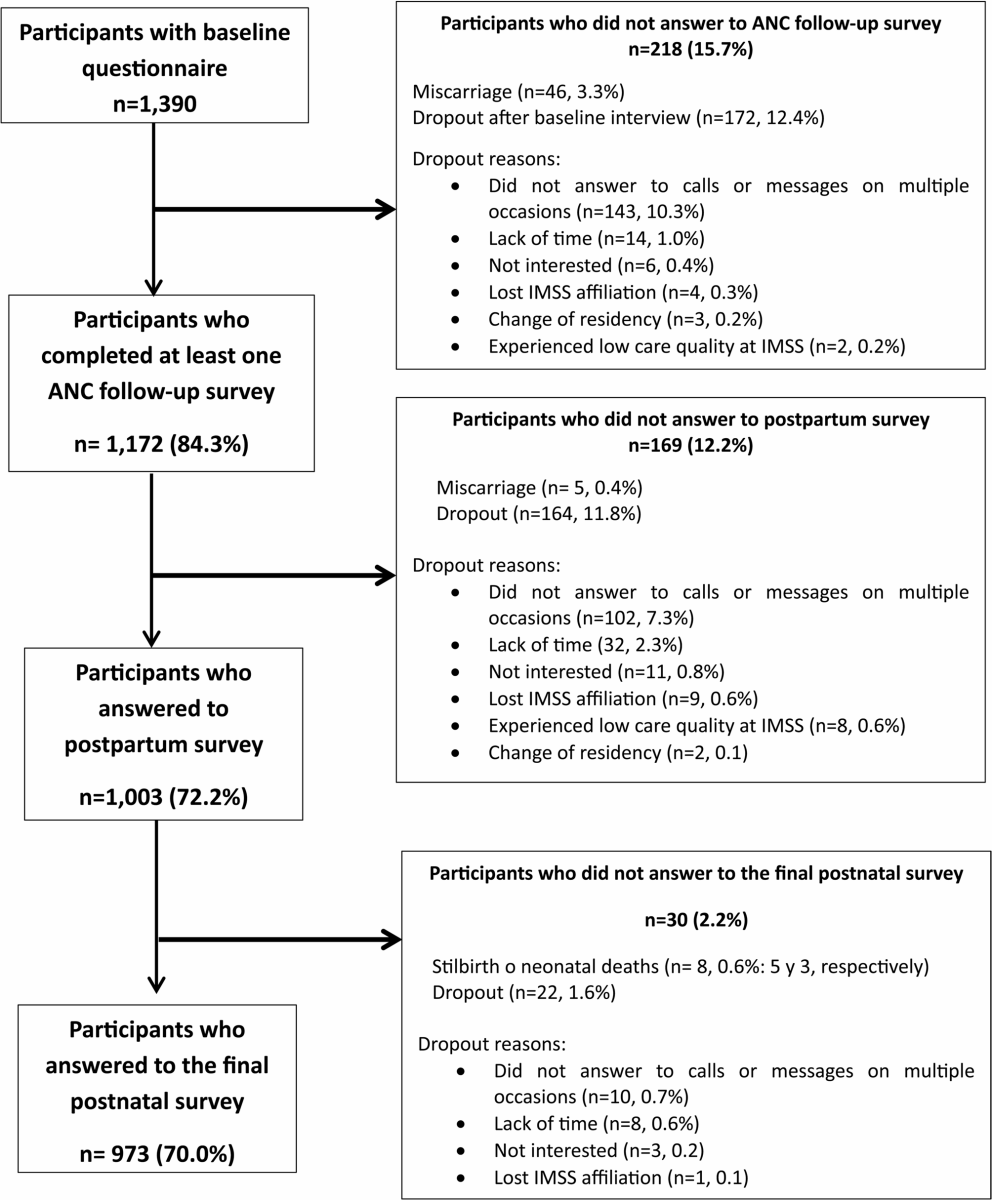
Flow of maternal eCohort participants (IMSS, Mexico 2023-2024)

Figure 1 depicts the flow of maternal eCohort participants. Of the 1,390 women who agreed to participate and completed the baseline questionnaire, 84.3% completed at least one antenatal care (ANC) follow-up survey interview. The median follow-up duration—from the start of ANC to the conclusion of pregnancy—was seven months (range: 1–9 months). Only 10.5% completed all monthly surveys from enrollment through the end of pregnancy, while 54.2% completed surveys at least every other month.

### Losses to follow-up and withdrawals

Overall, 3.3% of participants experienced a miscarriage, and 12.4% withdrew from the study (Fig. 1). Most dropouts occurred because participants repeatedly did not answer phone calls or messages (10.3%), while 1.4% cited lack of time or interest. Additional reasons included moving residence, loss of IMSS affiliation, or poor care experiences. Furthermore, 12.2% of participants did not respond to a postpartum survey (0.4% due to miscarriage and 11.8% withdrawal), resulting in 1,003 participants who completed the postpartum survey. Finally, 973 women (70.0%) completed the postnatal survey; of these, 0.6% (*n* = 8) experienced fetal or neonatal death and 1.6% (*n*=22) withdrew.

### Baseline characteristics by follow-up group

Additional Tables 1-3 compare baseline demographics, medical history, and first ANC visit attributes among four groups: (i) women who completed at least one ANC follow-up and the postpartum surveys and delivered at IMSS; (ii) women who completed the surveys but delivered at a private or another public healthcare provider; (iii) women who dropped out after the baseline interview; and (iv) women who dropped out during prenatal follow-up. Women who completed ANC follow-up surveys but delivered outside IMSS were more likely to have a university degree, be married, and identify as housewives, unemployed, or students. They also reported planned pregnancies more often and were primigravids. In contrast, women who dropped out after baseline were more often single, divorced, or separated, reported experiences of intimate partner violence, and had higher rates of pregnancy warning signs. At their first ANC visit, they rated the quality of care as poor or fair and more frequently described long waiting times, limited provider time, unclear explanations, poor involvement in decision-making, lack of respect, unhelpful staff, and inadequate medical equipment.

### Overall eCohort profile

After excluding 51 women who had miscarriages, the eCohort consisted of 1,339 women. Most of them were between 18 and 34 years old (86.9%), with high school or higher (64.9%), and had a paid job (60.6%) (Table 1). The majority (82%) lived in a common law union or were married; 9.5% reported suffering from some form of intimate partner violence. Only 4.9% reported consuming alcoholic beverages and/or smoking.

**Table 1:** Characteristics of pregnant women included in the eCohort study

Variable	Total *n*=1339
I. Demographic characteristics and risky health behaviors	*n* (%)
Age groups	
18–34 years	1164 (86.9)
≥35 years	175 (13.1)
Level of education	
With or without elementary school	62 (4.6)
Completed secondary school	406 (30.3)
High school or higher	869 (64.9)
No answer	2 (0.2)
Occupation	
Remunerated job	812 (60.6)
Housewife/unemployed/student	527 (39.4)
Marital status	
Married or partnered	1098 (82.0)
Single/divorced/separated/widow	218 (16.3)
Did not answer	23 (1.7)
Suffering some form of intimate partner violence	127(9.5)
Risky health behaviors (consuming alcoholic beverages and/or smoking)	65 (4.9)
II. Medical and obstetric history at baseline	*n*=1339
Initiation of antenatal care	
First trimester	592 (44.2)
Second trimester	585 (43.7)
Third trimester	162 (12.1)
Multigravida	842 (62.9)
History of miscarriage/stillborn baby	65 (4.9)
History of premature birth (<37 weeks of gestation)	65 (4.9)
Multiple pregnancy	16 (1.2)
History of pre-gestational chronic disease(s)	200 (14.9)
History of hypertension	26 (1.9)
History of diabetes	25 (1.8)
III. Health problems during pregnancy	
Urinary tract infection	381 (28.5)
Risk of depression	314 (23.5)
Threat of miscarriage/vaginal bleeding	222 (16.6)
Anemia	125 (9.3)
Hypertensive disorders	87 (6.5)
Gestational diabetes	61 (4.6)
IV. Duration and adherence to follow-up within the eCohort	
Duration of the follow-up in the eCohort, based on the start of the antenatal care and end of the pregnancy (months) median (minimum-maximum)	7 (1–9)
Adherence to the monthly survey interviews (with one monthly follow-up interview)	*n* (%) 140 (10.5)
≥50% adherence (at least one interview every 2 months)	726 (54.2)

### Antenatal care initiation and pregnancy history

Most women started ANC in the first (44.2%) or second trimester (43.7%). Nearly two-thirds (62.9%) had previous pregnancies, 4.9% reported a history of miscarriage or premature birth, and 1.2% had multiple pregnancies. Regarding medical history, 14.9% reported chronic illness before pregnancy (1.9% with hypertension; 1.8% with diabetes).

### Pregnancy-related complications

During the current pregnancy, the most frequent complications were urinary tract infections (28.5%), risk of depression (23.5%), threat of miscarriage (16.6%), anemia (9.3%), hypertensive disorders (6.5%), and gestational diabetes (4.6%).

### ANC visits and service utilization

The median number of ANC visits was six (range: 1–18) (Table 2). Only 36% met the WHO recommendation of eight visits. Additionally, 17.9% had consultations with an obstetrician, 51.7% received ANC follow-up at the referral hospital, and 5.5% also saw private providers. Among the 1,167 women who participated in at least one ANC follow-up survey, 69.2% used dental services, 10.5% attended consultations for acute or chronic conditions, 4.5% had virtual ANC consultations, and 28.6% sought emergency services. The main reasons for emergency visits were suspected miscarriage (43.1%), hypertensive disorders (17.4%), urinary or vaginal infections (10.5%), respiratory or gastrointestinal infections (8.1%), and other causes.

**Table 2:** Use of health services and content of antenatal care during pregnancy

Variable	Total *n*=1339
I. Use of health services during pregnancy	*n* (%)
Number of ANC visits per women,	
mean (SD)	6.5 (4.0)
median (minimum-maximum)	6 (1–18)
Compliance with 8 ANC consultations recommended by the WHO	482 (36.0%)
Consultation with an obstetrician	239 (17.9)
Pregnancy follow-up at the hospital	692 (51.7)
ANC visits with private provider during pregnancy	73 (5.5)
Virtual ANC consultations	*n*=1,167 52 (4.5)
Use of dental services	808 (69.2)
Consultations due to acute or chronic diseases	123 (10.5)
Visits to emergency services	334 (28.6)
Number of emergency services visits per woman.	
median (minimum-maximum)	0 (0–7)
II. Clinical activities and quality of care perceived by women during ANC	*n*=1339
Weight measurement at each ANC visit	1303 (97.3)
Blood pressure measurement at each ANC visit	1247 (93.1)
Blood test(s)	1321 (98.7)
Urine test(s)	1316 (98.3)
Ultrasound examination	1082 (80.8)
Warning signs counseling	1231 (91.9)
Nutrition counseling	1162 (86.8)
Birth preparedness counseling	*n*=1167 806 (69.1)
Newborn care counseling	810 (69.4)
Psychological counseling for women at risk of depression	*n*=314
Yes	12 (3.8)
No	121 (38.5)
Did not answer	181 (57.7)
Folic acid supplementation during the first trimester	*n*=592
No or only sometimes	143 (24.2)
Every visit	449 (75.8)
Iron supplementation starting from 20 weeks of gestation	*n*=1167
No or only sometimes	911 (78.1)
Every visit	251 (21.5)
Did not answer	5 (0.4)
Healthcare competence as a percentage of necessary clinical actions performed during ANC	*n*=1339
median (minimum-maximum)	81.8 (0–100)
Receiving ≥80% of necessary ANC activities	868 (64.8)
ANC care quality perceived by women	
Excellent/Very good/Good	938 (70.1)
Poor/Fair	401 (29.9)

### Content and quality of ANC

During ANC visits, over 90% reported weight and blood pressure checks at each appointment, as well as blood and urine tests. Ultrasound scans were performed in 80.8% of cases; 91.9% received counseling on warning signs; 86.8% received nutrition counseling (89.7% of women with hypertension; 96.7% of women with diabetes); and 69% received labor and newborn care guidance. However, only 3.8% of 314 women at risk for depression received psychological counseling. The median percentage of essential ANC actions completed was 81.8%, with 64.8% receiving ≥ 80% of recommended actions. Despite this, 30% rated at least one ANC visit as poor or fair quality (Table 2).

### Factors associated with ANC utilization

Table 3 shows the factors associated with the number of ANC visits. Characteristics associated with higher ANC attendance included suffering from hypertensive disorders of pregnancy (aIRR 1.08; 95% CI:1.01, 1.15), urinary tract infection (aIRR 1.08; 95% CI:1.05, 1.13) or anemia (aIRR 1.09; 95% CI:1.03, 1.16), initiating antenatal care in the first or second trimester (aIRR 2.01; 95% CI:1.82, 2.22 and aIRR 1.49; 95% CI:1.36, 1.64, respectively), having a consultation with an obstetrician (aIRR 1.36; 95% CI:1.24, 1.49), or ANC follow-up at the hospital (aIRR 1.32; 95% CI:1.23, 1.41), and receiving ANC in either the northern (aIRR 1.66; 95% CI:1.45, 1.89) or southeastern (aIRR 1.30; 95% CI:1.13, 1.49) regions.

**Table 3:** Factors associated with the number of antenatal care visits (IP-weighted Negative binomial multivariable regression analysis, *n*=1160)

Variable	Adjusted IRR	Clustered Robust Std. Err	95%CI	*p*
I. Sociodemographic characteristics and medical history				
Age ³35 years	1.04	0.03	0.98, 1.11	0.223
Level of education				
With or without elementary school	1.01	0.05	0.91, 1.11	0.907
Completed secondary school	1.04	0.02	0.99, 1.09	0.060
Single/divorced/separated/widow	0.99	0.03	0.93, 1.07	0.950
Suffering some type of intimate partner violence	0.95	0.04	0.86, 1.04	0.260
Remunerated job	1.02	0.03	0.97, 1.08	0.449
Risky health behaviors	0.95	0.04	0.87, 1.03	0.226
Multigravida	0.99	0.02	0.94, 1.04	0.621
History of miscarriage/stillborn baby	1.02	0.06	0.91, 1.14	0.764
History of premature birth (<37 weeks of gestation)	1.03	0.05	0.93, 1.13	0.597
History of pre-gestational chronic disease(s)	0.97	0.03	0.91, 1.03	0.294
Hypertensive disorders of pregnancy	1.08	0.03	1.01, 1.15	0.020
Gestational diabetes during pregnancy	0.99	0.06	0.88, 1.11	0.846
Urinary tract infection during pregnancy	1.08	0.02	1.05, 1.13	<0.001
Anemia during pregnancy	1.09	0.03	1.03, 1.16	0.003
Threat of miscarriage/vaginal bleeding during pregnancy	1.02	0.03	0.96, 1.07	0.550
Risk of depression during pregnancy	0.96	0.02	0.91, 1.01	0.124
II. Use of health services during pregnancy				
Initiation of antenatal care in:				
First trimester	2.01	0.10	1.82, 2.22	<0.001
Second trimester	1.49	0.07	1.36, 1.64	<0.001
ANC visits with private provider during pregnancy	1.09	0.06	0.98, 1.21	0.109
Consultation with an obstetrician	1.36	0.06	1.24, 1.49	<0.001
Pregnancy follow-up at the hospital	1.32	0.05	1.23, 1.41	<0.001
ANC care perceived as poor or fair quality	1.001	0.03	0.95, 1.05	0.969
III. Location and size of the family medicine clinic where the woman received antenatal care				
Region				
North	1.66	0.11	1.45, 1.89	<0.001
Southeast	1.30	0.09	1.13, 1.49	<0.001
West	1.03	0.04	0.95, 1.11	0.473
Size of the clinic				
Medium	1.04	0.08	0.89, 1.21	0.624
Large	1.02	0.08	0.88, 1.18	0.778

Dependent variable: Number of ANC visits. Reference categories: age: under 35 years; education: high school or higher; marital status: married; intimate partner violence: none reported; employment: unemployed; health behaviors: no risky behaviors; pregnancy history: primigravida; no miscarriages, no stillbirths, or premature births; current health conditions: no chronic diseases, no hypertensive disorders, no gestational diabetes, no urinary tract infections, or anemia; no threat of miscarriage or vaginal bleeding; no depression risk; antenatal care: initiated in the third trimester; no private provider ANC visits; no consultation with an obstetrician; no pregnancy follow-up at the hospital; quality of ANC perceived as good/very good or excellent; region: central region; clinic type: small clinic

### Factors associated with emergency services use

Table 4 shows the factors associated with the number of emergency department visits during pregnancy. Characteristics associated with emergency department use during pregnancy included preexisted chronic diseases (aIRR 1.52; 95% CI:1.21, 1.91), hypertensive disorders of pregnancy (aIRR 1.85; 95% CI:1.15, 2.98), urinary tract infections (aIRR 1.30; 95% CI:1.02, 1.62), the threat of miscarriage (aIRR 1.73; 95% CI:1.14, 2.61), or risk of depression (aIRR 1.50; 95% CI:1.12, 2.01), as well as receiving ANC in the central region (aIRR 1.76; 95% CI:1.24, 2.49) and medium-size family medicine clinic (aIRR 1.94; 95% CI:1.05, 3.59).

**Table 4:** Factors associated with the number of emergency services visits during pregnancy (IP-weighted negative binomial multivariable regression analysis, *n* = 1160)

Variable	Adjusted IRR	Clustered Robust Std. Err	95%CI	*p*
I. Sociodemographic characteristics and medical history				
Age ³35 years	0.99	0.15	0.73, 1.34	0.939
Level of education				
With or without elementary school	1.20	0.44	0.58, 2.47	0.629
Completed secondary school	1.04	0.14	0.80, 1.36	0.762
Single/divorced/separated/widow	0.90	0.13	0.67, 1.20	0.480
Suffering some type of intimate partner violence	0.79	0.17	0.52, 1.21	0.283
Remunerated job	1.23	0.16	0.95, 1.59	0.111
Risky health behaviors	1.12	0.30	0.67, 1.90	0.660
Multigravida	0.90	0.10	0.72, 1.13	0.364
History of miscarriage/stillborn baby	0.88	0.21	0.55, 1.41	0.589
History of premature birth (<37 weeks of gestation)	0.90	0.25	0.52, 1.54	0.693
History of pre-gestational chronic disease(s)	1.52	0.18	1.21, 1.91	<0.001
Hypertensive disorders of pregnancy	1.85	0.45	1.15, 2.98	0.011
Gestational diabetes during pregnancy	1.26	0.31	0.78, 2.03	0.351
Urinary tract infection during pregnancy	1.30	0.15	1.02, 1.62	0.019
Anemia during pregnancy	1.07	0.17	0.77, 1.47	0.693
Threat of miscarriage/vaginal bleeding during pregnancy	1.73	0.36	1.14, 2.61	0.009
Risk of depression during pregnancy	1.50	0.22	1.12, 2.01	0.006
II. Use of health services during pregnancy				
Initiation of antenatal care in				
First trimester	1.43	0.39	0.84, 2.43	0.185
Second trimester	1.16	0.26	0.72, 1.78	0.580
ANC visits with private provider during pregnancy	0.89	0.23	0.54, 1.47	0.646
Consultation with an obstetrician	1.22	0.29	0.77, 1.93	0.404
Pregnancy follow-up at the hospital	1.36	0.23	0.98, 1.88	0.065
Healthcare competence as a percentage of activities performed during ANC	0.99	0.004	0.99, 1.003	0.216
ANC care perceived as poor or fair quality	1.03	0.13	0.80, 1.33	0.812
III. Location and size of the family medicine clinic where the woman received antenatal care				
Region				
Central	1.76	0.31	1.24, 2.49	0.002
West	1.18	0.20	0.84, 1.64	0.339
South	1.07	0.23	0.70, 1.63	0.761
Size of the clinic				
Medium	1.94	0.61	1.05, 3.59	0.034
Large	1.53	0.47	0.84, 2.81	0.165

Dependent variable: use of emergency services during pregnancy. Reference categories: age: under 35 years; education: high school or higher; marital status: married; intimate partner violence: none reported; employment: unemployed; health behaviors: no risky behaviors; pregnancy history: primigravida; no miscarriages or stillbirths, no premature births; current health conditions: no chronic diseases, no hypertensive disorders, no gestational diabetes, no urinary tract infections, or anemia; no threat of miscarriage or vaginal bleeding; no depression risk; antenatal care: initiated in the third trimester; no private provider ANC visits; no consultation with an obstetrician; no pregnancy follow-up at the hospital; healthcare competence as a percentage of activities performed during ANC (continuous variable); quality of ANC perceived as good/very good or excellent; region: north region; clinic type: small clinic

## Discussion

This study presents findings from a prospective cohort of pregnant women who received ANC at IMSS. We found that women attended an average of six ANC consultations throughout pregnancy, with nearly 65% receiving 80% or more of the recommended care content. Psychological counseling for depression and counseling on labor and newborn care were frequently omitted. Almost 30% of women who completed pregnancy follow-up rated at least one ANC visit as poor or fair. Higher ANC use was associated with hypertensive disorders of pregnancy, urinary tract infection, anemia, early ANC initiation, consultations with obstetricians, hospital-based follow-up, and residence in northern or southeastern regions. In contrast, greater use of emergency services was linked to preexisting chronic diseases, hypertensive disorders, and risk of miscarriage or depression.

The WHO recommends a minimum of eight ANC contacts that prioritize respectful, woman-centered care to reduce perinatal mortality and improve maternal experience [30]. This is based on evidence that four-visit ANC models are linked to higher perinatal mortality, particularly in low-resource settings [45], and that more frequent ANC consultations are associated with greater maternal satisfaction when care is respectful and culturally appropriate [46]. In our study, the median number of ANC visits was six, with only 36% of women meeting the WHO eight-visit target. This is higher than the 9% reported across 14 sub-Saharan African countries [47] and the 13% found in a multi-country study spanning Africa, Asia, the Middle East, Europe, and Oceania [48].

Three in ten women in our cohort perceived ANC quality as poor or fair, and many did not receive the full set of 12 essential clinical activities. This aligns with a recent systematic review showing that more than two-thirds of ANC users worldwide receive substandard care [49]. The combination of moderate ANC attendance and persistent quality gaps highlights the need for ongoing training of IMSS healthcare providers on WHO and IMSS ANC guidelines. Training should emphasize the content of ANC, effective communication, and respectful care. Such efforts could improve provider competence, promote adherence to clinical standards, and strengthen continuity of care.

Urinary tract infections (UTIs) were frequent, affecting 28.5% of participants—higher than the 11% reported in several African countries [50] and consistent with rates in Latin America [51]. Women with UTIs were more likely to use emergency departments and reported a higher number of ANC visits. Given UTIs’ role in increasing the risk of preeclampsia [52], preterm birth, and low birth weight, regular screening for bacteriuria and timely antimicrobial treatment are warranted [51, 53].

Risk of depression was the second most common condition (23.5%), yet only 3.8% of at-risk women received psychological counseling. Depression during pregnancy increases the likelihood of preterm birth and low birth weight [54, 55], and in this study, it was associated with increased emergency service use. While the American College of Obstetricians and Gynecologists recommends depression screening in each trimester [56], IMSS guidelines only suggest preventive measures for women with “known risk factors” without specifying these factors. This lack of clarity likely hinders systematic screening and management.

Anemia affected 9.3% of pregnant women in the eCohort. During pregnancy, anemia can contribute to low birth weight, preterm birth, and increased risks of both perinatal and neonatal mortality [57]. In low- and middle-income countries, maternal anemia is linked to 12% of low-birth-weight cases, 19% of preterm births, and 18% of perinatal deaths. To combat anemia, WHO recommends daily iron supplementation, while IMSS guideline advises starting this supplementation at 20 weeks of gestation and continuing until delivery. However, only 21.5% of women in the eCohort reported receiving iron prescriptions at every ANC consultation.

Hypertension disorders were present in 6.5% of the eCohort, which aligns with the WHO report indicating that hypertension disorders affect 5–10% of pregnancies [58]. These conditions significantly increase risks of maternal mortality and preterm birth [59]. In our study, hypertensive disorders were associated with greater use of both ANC and emergency services, likely reflecting challenges in blood pressure control. This underscores the importance of closely monitoring women with hypertension or related risk factors and recommending lifestyle modifications, such as adopting a Mediterranean diet and engaging in moderate physical activity [59]. Notably, although nutritional counseling is critical for managing hypertension, 1 in 10 hypertensive women in our cohort reported not receiving it.

Neural tube defects remain a concern in Mexico, with rates of 11–34 per 10,000 births [60]. Folic acid supplementation before conception and during the first trimester can markedly reduce this risk [61]. In this study, 75.8% of women reported receiving folic acid in the first trimester, below the 90.2% coverage reported in ENSANUT 2022 [33]. Expanding micronutrient supplementation—ideally through multiple micronutrient formulations—could be a cost-effective strategy to meet the nutritional needs of pregnant women, especially in low- and middle-income countries [62].

Globally, the use of emergency services by pregnant women varies widely by country, ethnicity, and disability, with utilization rates ranging from 15% to 90% [20, 63, 64] and linked to severe maternal morbidity [65]. In this study, nearly three in ten women used emergency services during pregnancy, most often for signs of possible miscarriage or conditions such as hypertensive disorders, infections, or risk of depression. These findings underscore the need to prioritize women with such health issues during routine ANC.

Our study has limitations. First, participants self-reported the care they received, which may lead to inaccuracies about reported clinical activities provided by the health professionals. Also, more educated or multiparous women might recall care details better. Social desirability bias may influence participants’ responses; however, conducting interviews via phone surveys, where the interviewer and participants are separated, can encourage more honest responses. Lastly, we faced significant missing data due to 12.4% dropout rate during the antenatal follow-up, this is similar to the 12% seen in the maternal and newborn health eCohort studies conducted in Ethiopia, India, Kenya, and South Africa [66]. These studies achieved this follow-up rates by providing participants with phones and monthly airtime; those who opted out of receiving a phone were given equivalent airtime instead. Regrettably, our study lacked the financial resources to provide such incentives. To mitigate potential bias from missing data due to dropout, we employed stabilized inverse probability weights.

In conclusion, strengthening ANC at IMSS is essential to improve women’s experiences and encourage greater service utilization. Key opportunities include enhancing provider competence in woman-centered care; expanding counseling on nutrition, psychological well-being, birth preparedness and newborn care; ensuring iron and folic acid supplementation; and improving the identification and management of women with chronic diseases, pregnancy complications, or risk of depression.

Future research should continue to integrate women’s perspectives and evaluate effectiveness of interventions aimed at improving access to and quality of antenatal care. For instance, this research could embed women’s preferences into new care models, such as telemedicine options for virtual pregnancy consultations, education, and monitoring—particularly for those who face challenges like transportation and other obstacles to attending in-person appointments. Special attention should be given to vulnerable groups, including pregnant adolescents, women with chronic diseases, those facing high-risk conditions like preeclampsia and hemorrhage, and women experiencing intimate partner violence.

## Supplementary Information

Supplementary Material 1.

## Data Availability

The datasets analysed during the current study are available from the corresponding author on reasonable request.

## Abbreviations

ANC: antenatal care
ED: emergency department
IMSS: Mexican Institute of Social Security
IPW: inverse probability weights
LA: Latin America
OECD: Organisation for Economic Co-operation and Development
PHQ-9: Patient Health Questionnaire
WHO: World Health Organization
