# Supplementary material for: Factors influencing pregnant women’s use of antenatal and emergency care services covered by social security: findings from the maternal eCohort in Mexico
Source: BMC Pregnancy Childbirth. 2025 Nov 4;25:1160. doi: 10.1186/s12884-025-08301-9 (PMC12584554; doi:10.1186/s12884-025-08301-9)
Supplement: Supplementary file 1 — Supplementary Material 1. [file 12884_2025_8301_MOESM1_ESM.pdf]

Additional file 1

Additional Table 1. Comparison of demographic characteristics, obstetric and medical history of the IMSS maternal eCohort participants who completed the eCohort activities and those who dropped out

Variable	Completed the postpartum survey and delivery was at IMSS facility	Completed the postpartum survey and delivery was at a service other than IMSS	Dropout after baseline survey interview	Dropout after completing at least one ANC follow-up survey	p
	n=841	n=162	n=172	n=164	
**I. Demographic characteristics and risky health behaviors**	n (%)	n (%)	n (%)	n (%)	
**Age groups**					
18-34 years	728 (86.6)	135 (83.3)	153 (89.0)	148 (90.2)	0.013
≥35 years	113 (13.4)	27 (16.7)	19 (11.0)	16 (9.8)	
**Level of education**					
With or without elementary school	39 (4.6)	6 (3.7)	7 (4.1)	10 (6.1)	
Completed secondary school	285 (33.9)	20 (12.4)	51 (29.6)	50 (30.5)	<0.01
High school	352 (41.9)	53 (32.7)	71 (41.3)	70 (42.7)	
University degree	164 (19.5)	83 (51.2)	42 (24.4)	34 (20.7)	
Did not answer	1 (0.1)	0 (0)	1 (0.6)	0 (0)	
**Occupation**					
Housewife/unemployed/student	355 (42.2)	42 (25.9)	61 (35.5)	69 (42.1)	<0.01
Paid job	486 (57.8)	120 (74.1)	111 (64.5)	95 (57.9)	
**Risky health behaviors**	42 (5.0)	7 (4.3)	9 (5.2)	7 (4.3)	0.086
Alcohol consumption	31 (3.7)	3 (1.9)	7 (4.1)	5 (3.1)	0.062
Tobacco consumption	15 (1.8)	4 (2.5)	2 (1.2)	2 (1.2)	0.840
**Marital status**					
Partnered	385 (45.8)	60 (37.0)	70 (40.7)	62 (37.8)	
Married	329 (39.1)	74 (45.7)	62 (36.1)	56 (34.2)	<0.001
Single/divorced /separated/widow	122 (14.5)	26 (16.1)	38 (22.0)	32 (19.5)	
Did not answer	5 (0.6)	2 (1.2)	2 (1.2)	14 (8.5)	

**Suffering some type of intimate partner violence**	71 (8.4)	7 (4.3)	21 (12.2)	28 (17.1)	<0.001
**II. Obstetric history**					
Primigravida	288 (34.2)	80 (49.4)	67 (39.0)	62 (37.8)	0.001
**Among women with a previous pregnancy:**	n=553	n=82	n=105	n=102	
Miscarriage/stillborn baby	49 (8.9)	4 (4.9)	5 (4.8)	7 (6.9)	0.046
Premature birth (<37 weeks of gestation)	45 (8.1)	6 (7.3)	7 (6.7)	7 (6.9)	0.980
Obstetric hemorrhage	22 (4.0)	3 (3.7)	3 (2.9)	2 (2.0)	0.572
Cesarean section	202 (36.5)	40 (48.8)	47 (44.8)	38 (37.3)	0.069
**III. Medical history**					
History of pre-gestational chronic disease(s)	145 (17.2)	35 (21.6)	34 (19.8)	35 (21.3)	0.156
**Five most frequent diseases**					
Depression/anxiety	38 (4.5)	14 (8.6)	9 (5.2)	12 (7.3)	0.119
Hypertension or cardiovascular disease	19 (2.3)	4 (2.5)	2 (1.2)	9 (5.5)	0.059
Diabetes/prediabetes/insulin resistance	19 (2.3)	0 (0)	5 (2.9)	1 (0.6)	0.008
Thyroid disease	22 (2.6)	5 (3.1)	4 (2.3)	3 (1.8)	0.873
Gynecological disease	15 (1.8)	4 (2.5)	3 (1.7)	5 (3.1)	0.260

Additional Table 2. Comparison of current pregnancy characteristics and health status reported at the baseline survey by the IMSS maternal eCohort participants who completed the eCohort activities and those who dropped out

Variable	Completed the postpartum survey and delivery was at IMSS facility	Completed the postpartum survey and delivery was at a service other than IMSS	Dropout after baseline survey interview	Dropout after completing at least one ANC follow-up survey	p
	n=841	n=162	n=172	n=164	
**I. Current pregnancy**	n (%)	n (%)	n (%)	n (%)	
Initiation of antenatal care					
First trimester	356 (42.3)	73 (45.1)	94 (54.7)	69 (42.1)	
Second trimester	379 (45.1)	73 (45.1)	62 (36.0)	71 (43.3)	<0.001
Third trimester	106 (12.6)	16 (9.8)	16 (9.3)	24 (14.6)	
Planned pregnancy	n=841	n=162	n=172	n=164	
	380 (45.2)	104 (64.2)	86 (50.0)	80 (48.8)	<0.001
Multiple pregnancy	n=689	n=126	n=145	n=127	
	7 (1.0)	3 (2.4)	3 (2.1)	3 (2.4)	0.364
**Warning sign reported at the first ANC visit**	n=841	n=162	n=172	n=164	
Number of emergency warning signs					
0	554 (65.9)	106 (65.4)	112 (65.1)	90 (54.9)	
1	189 (22.5)	36 (22.2)	31 (18.0)	44 (26.8)	0.068
≥2	98 (11.6)	20 (12.4)	29 (16.9)	30 (18.3)	
Presence of one or more obstetric risk factors[‡]	227 (27.0)	42 (25.9)	42 (24.4)	40 (24.4)	0.045
**II. Health status**					
**Self-rated health**					
Fair/Poor	141 (16.8)	18 (11.1)	29 (16.9)	23 (14.0)	0.034
**Risk of depression**					
Minimal risk 0-4	697 (82.9)	134 (82.7)	136 (79.1)	123 (75.0)	
Mild risk 5-9	126 (14.9)	20 (12.4)	27 (15.7)	32 (19.5)	0.021
Moderate to severe ≥10	19 (2.2)	8 (4.9)	9 (5.2)	9 (5.5)	

Additional Table 3. Comparison of healthcare competency and quality of care perceived by women at the first antenatal care visit reported at the baseline survey by the IMSS maternal eCohort participants who completed the eCohort activities and those who dropped out

Variable	Completed the postpartum survey and delivery was at IMSS facility	Completed the postpartum survey and delivery was at a service other than IMSS	Dropout after baseline survey interview	Dropout after completing at least one ANC follow-up survey	p
	n=841	n=162	n=172	n=164	
	n (%)	n (%)	n (%)	n (%)	
**Healthcare competence (% of clinical activities performed from those required)**<br>Mean (SD)<br>Median (minimum-maximum)	68.6 (12.8)<br>70<br>(23.8-100)	67.7 (14.0)<br>68.2<br>(28.6-100)	66.4 (14.1)<br>66.7<br>(28.6-95.7)	65.5 (14.4)<br>66.0<br>(12-100)	0.009
**Perceived quality of care during the first ANC**					
**Waiting time**<br>Poor/Fair	426 (50.7)	85 (52.5)	88 (51.2)	94 (57.3)	0.002
**Time the provider spent with the woman**<br>Poor/Fair	368 (43.8)	68 (42.0)	76 (44.2)	84 (51.2)	0.001
**Clarity of the provider's explanations**<br>Poor/Fair	304 (36.2)	57 (35.2)	56 (32.6)	69 (42.1)	0.001
**Degree to which the provider involved women in decisions about their care**<br>Poor/Fair	333 (39.6)	65 (40.1)	63 (36.6)	76 (46.3)	0.032
**Knowledge and skills of their healthcare provider**<br>Poor/Fair	342 (40.7)	60 (37.0)	68 (39.5)	76 (46.3)	0.001

**Courtesy and helpfulness of the healthcare facility staff, other than healthcare provider**<br>Poor/Fair	356 (42.3)	71 (43.8)	63 (36.6)	75(45.7)	0.004
**Level of respect showed by the provider**<br>Poor/Fair	253 (30.1)	44 (27.2)	48 (27.9)	59 (36.0)	0.003
**Availability of medical equipment or access to lab tests**<br>Poor/Fair	438 (52.1)	94 (58.0)	81 (47.1)	94 (57.3)	0.001
**A summative score of women perception of the first ANC**<br>(Mean (SD)<br>Median (minimum-maximum)	23.2 (6.9)<br>23 (8-40)	23.2 (6.5)<br>23 (8-40)	22.7 (7.2)<br>23 (9-40)	22.0 (6.7)<br>21 (11-38)	0.016
